# Effective risk governance requires risk communication experts

**DOI:** 10.4178/epih.e2016055

**Published:** 2016-12-02

**Authors:** Hye-Jin Paek

**Affiliations:** Department of Advertising & Public Relations, Hanyang University, Ansan, Korea

There was a lot of discussion about communication at the lecture seminar, and it was quite surprising. Among several lessons left by the Middle East Respiratory Syndrome (MERS) epidemic last year, the most significant one is the realization of the importance of communication in a crisis situation.

Unlike scientists and experts who recognize risk based on scientific evidence, the general population tends to have more fear and perceive more risk than the actual risk itself due to uncertainties created by insufficient and inaccurate information. Therefore, risk communication plays a major role in reducing the gap between real and perceived risk, and in effectively delivering accurate information. As a result of the failure of risk communication about MERS, the social and economic disturbance was enormous, and the importance of risk communication was realized in other fields such as healthcare. This is one of the outcomes of experiencing the severity of the MERS epidemic.

But as an expert in health communication, I think the concept of communication is only superficially understood. There is a tendency to think of it only as determining “what” to say. However, communication is a complex process that requires not only consideration of content, but also consideration of how, to whom, at what point, and through which channel to deliver the content. Risk communication must also consider the nature of risk, people’s characteristics affecting their risk perceptions, and the complex media environment. Thus, communication experts should be involved with health and medical professionals from the very beginning of the response to a public health crisis. At the time of the initial response to MERS, risk communication was focused on “what” health professionals thought should be said to the public. It was unfortunate that risk communication experts were not involved in the process until much later.

After the risk communication failure associated with MERS, the government set up in the Disease Control Headquarters a communication room and a communication officer modeled after the communication systems of the US Centers for Disease Control and Prevention. Early this year, when the Zika virus spread in South America, the Communications Office quickly set up a civilian communication advisory board and actively responded to the press as well as to the public. Such an action goes hand-in-hand with the arguments that the government should be more aggressive in communicating risks. However, no matter how actively the press and other people communicate, their communication will not be effective unless the government can be trusted. Building trust is a difficult task that takes a long time, but it takes one second for trust to be lost. Through the MERS epidemic, we witnessed the collapse of public trust in government. In fact, Lee et al.’s research [1] demonstrated that the overall confidence in the government response to the MERS epidemic was 15.6%, which was comparatively less than the 24% reported during the 2009 H1N1 flu epidemic. In crisis situations, the public tends to ignore information and guidelines presented by a distrusted government. In a serious public health crisis such as an infectious disease epidemic, public distrust of the government is a concern that can worsen confusion by causing neglect of or resistance to important government actions, such as an imposing isolation or prioritizing distribution of necessities. However, the results of Lee et al.’s research [1] were unexpected, as they did not show any significant relationship between trust in government, preventive action, and infection sensitivities of residents during the MERS epidemic. Perhaps this result was due to self-directive preventive action such as wearing a mask. If the preventive action consisted of government-imposed guidelines or recommendations such as quarantines, prohibition of movement, and distribution of resources, then there might have been significant relationships between the three variables.

Another point to keep in mind is that the stakeholders comprising the “who” in risk communication are far more diverse than commonly assumed. In risk communication, stakeholders usually tend to be divided into four broad categories: government, professional experts, people, and media. However, in reality, there are more diverse groups of stakeholders within each of these four categories, and we must consider the conflicts and differences that may be present among these groups. For example, the fact that the central control tower and Seoul city had a conflict over information disclosure at the time of the MERS epidemic proves this point.

There was considerable disagreement and confusion among healthcare professionals about the necessary responses to MERS, for example whether to inform the public about which areas were infected with MERS or which hospitals had MERS patients. In hospitals, there was also a conflict between management and medical staff (doctors, nurses, etc.). This has been vividly proven through the experience of the panelist who was at the Samsung Seoul Hospital at the time of MERS epidemic. Therefore, it is necessary to discuss strategies for reducing and resolving conflicts among various stakeholders in the context of a public health crisis. Compared to the past, when only three major broadcasters and four daily newspapers dominated the press, nowadays there is more competition in the news among various online media and social media.

In addition, the people who formerly only passively received information provided by broadcasts and newspapers are now playing the roles of “producers” and “disseminators” who reprocess and spread news through various social media. In this context, the boundaries of information disclosure and communication, which we formerly thought about only from the perspective of health care, should be redefined. There is no guarantee that information will be withheld even if a health care professional decides not to disclose it. Rather, the key concern now is that public anxiety will increase as a result of inaccurate information and rumors being spread on social media.

Lastly, there have been many important discussions about people’s trauma in the outbreak of infectious diseases. In particular, social support is an important concept among stakeholders in a crisis situation, and appropriate intervention and evaluation in response to crises are important research tasks. This issue is not only whether there was adequate social support but also what kind of support was provided, whether the content of that support was informational or emotional, and which aspect of that support was satisfactory. More detailed studies on these issues are urgently needed. Various public health crises will continue to arise in the future, and preparing for them is not the exclusive duty of public health and healthcare professionals. A more effective risk governance system that balances the three areas of risk assessment, risk management, and risk communication needs to be established. Such a system could help public health officials, in cooperation with public health professionals and risk experts, respond appropriately to actual and perceived risks.
