# Supplementary material for: Effective risk governance requires risk communication experts
Source: Epidemiol Health. 2016 Dec 2;38:e2016055. doi: 10.4178/epih.e2016055 (PMC5177806; doi:10.4178/epih.e2016055)
Supplement: Supplementary file 1 [file epih-38-e2016055-supplementary.pdf]

효과적인 위험 통제 시스템에는 위험 소통 전문가가 필요하다

한양대학교 광고홍보학과
백혜진

역학회 세미나에서 소통에 대해 많은 논의가 있어서 놀라웠다. 작년 메르스 사태가 남긴 여러 가지 교훈 중 위험 위기 상황에서의 소통의 중요성을 깨닫게 된 것이 가장 큰 소득이 아닐까 생각된다. 과학적인 증거를 기반으로 위험을 인식하는 과학자나 전문가와는 달리 일반인은 위험 자체의 불확실성 혹은 부족하고 부정확한 정보로 인한 불확실성으로 인해 실제 위험보다 위험을 더 크게 지각하고 두려워하는 경향이 있다. 따라서 위험 소통은 실제 위험과 지각된 위험의 차이를 줄이고 정확한 정보를 효과적으로 전달하는 데 큰 역할을 담당한다. 메르스 위험 소통의 실패로 인해 사회적 경제적 파장은 엄청났고, 이를 보건 의료 등 다른 분야에서도 깨닫게 된 것은 뼈아픈 메르스 사태를 경험함으로써 얻은 하나의 성과가 아닐까 생각된다.

다만, 헬스 커뮤니케이션 전공자의 입장에서 보면, 소통이 매우 피상적으로 이해되고 있다는 생각이 든다. 즉 소통이 “무엇”을 말할지를 결정하면 되는 것으로 이해하는 경향이 있다. 그러나 소통은 내용(content)뿐만 아니라 그 내용을 어떻게, 누구에게, 어느 시점에서, 어느 채널을 통해 전달할 것인가를 모두 고려해야 하는 복잡한 과정이다. 위험 소통은 또한 위험의 성격이나 수용자의 성향, 복잡한 미디어 환경 등을 고려해야 하기에 공중보건 위기 상황에 대응하는 초기부터 소통전문가가 보건, 의료전문가와 함께 투입되어야 한다. 메르스 초기 대응 당시에 위험 소통은 보건 의료 전문가 위주로 “무엇”을 공중에게 말할 것인가에 집중되어 있었고, 위험 소통 전문가가 늦게 투입된 점은 매우 아쉬운 점이다.

메르스의 뼈아픈 소통 실패 이후에 정부는 소통담당실과 소통담당관을 질병관리본부 안에 설치하고 미국 질병관리본부의 소통 체계 등을 벤치마킹 하는 등 노력을 지속하고 있다. 남미에서 지카바이러스가 확산된 올해 초 소통담당실에서는 재빠르게 민간소통자문단을 꾸리고 언론홍보는 물론 대국민 소통을 위해 적극적으로 대처했다. 이 점은 한 토론자가 정부가 커뮤니케이션을 적극적으로(aggressive) 해야 한다고 주장하신 내용과 상통하는 부분이다. 그러나 아무리 적극적인 언론 및 대국민 소통을 한다고 하더라도 정부 신뢰가 확보되지 않는다면 효과가 크지 않을 것이다. 신뢰는 구축하기는 힘들고 구축하는데 오랜 시간이 걸리는 어려운 작업인데 반해 무너지는 건 한순간이다. 우리는 메르스 사태를 통해 정부의 신뢰가 땅에 떨어지는 것을 목격했다. 실제로 이순영 교수님의 발표에서 메르스 유행시 전반적인 정부 대응에 대한 신뢰도는 15.6%로 2009년 신종플루 때의 24%에도 못 미치는 결과를 보였다. 위험상황에서 신뢰가 없는 정부가 제공하는 정보나 행동지침은 국민에게 외면 받는 경향이 있다. 정부에 대한 국민의 불신은 감염병과 같은 심각한 공중보건 위기 상황에서 격리나 구호품의 우선 배급 등 중요한 정부의 대처방안에 대한 무시나 저항을 야기함으로써 혼란을 가중시킬 수 있기에 우려가 되는 부분이다. 다만 이순영 교수님 연구 결과에서 메르스 유행시 지역사회 주민의 정부 신뢰도, 예방행위, 감염 민감도 변수 간에 유의미한 결과가 나오지 않았던 것은 뜻밖의 결과다. 아마도 예방행위가 마스크 착용 등 자기 주도의 행위였기 때문이었을 것이라 생각된다.

만약 예방행위가 격리나 이동 금지, 자원 분배 등 정부의 지침이나 권고행위였다면 유의미한 관계가 있지 않았을까 생각된다.

또 하나 고려할 점은 위험 위기소통에서 “누구”에 해당하는 이해관계자가 생각보다 훨씬 다양하다는 것이다. 위기 소통에서 이해관계자는 보통 정부, 전문가집단, 국민, 언론 크게 네 가지로 나누는 경향이 있다. 그러나 사실은 이 네 가지 축 각각의 내부에서 더 다양한 이해관계자가 있으며 이들 간의 갈등과 이견을 고려해야 한다. 예를 들어 메르스 당시 정보공개와 관련하여 중앙컨트롤타워와 서울시가 갈등했던 사례는 이 점을 입증한다. 보건의료 전문가사이에서도 메르스에 어떻게 대응해야 하는지 메르스의 감염지역이나 병원을 공개해야 하는지 등에 대해서 상당한 이견과 혼선이 있었으며 병원조직 내에서도 경영자와 의료인(의사, 간호사 등) 간의 갈등이 있었다. 이는 메르스 당시 삼성서울병원 현장에 계셨던 토론자의 경험담을 통해서도 생생하게 입증되고 있다. 따라서 공중보건 위기 상황에서 다양한 이해관계자간의 이견과 갈등을 줄이고 해결하는 전략 방안에 대한 논의도 필요하다. 언론만 보더라도 예전에 3대 방송사와 4대 일간지가 독점하다시피 하던 과거에 비해 현재는 다양한 온라인 미디어와 소셜미디어가 난립하면서 뉴스 경쟁이 과열되고 있다. 또한 과거 방송과 신문 뉴스를 수동적으로 받아들이기만 했던 “수용자”들은 이제 다양한 소셜 미디어를 통해 뉴스를 재가공하고 확산하는 뉴스의 “생산자” 이자 “확산자”의 역할을 동시에 하고 있다. 이런 상황에서 과거 보건의료관점에서 생각하던 정보 공개나 소통의 경계는 현재 상황에 맞게 재정의되어야 한다. 보건의료 전문가가 정보미공개를 결정했다고 해서 그 정보가 공개되지 않으리라는 보장이 없기 때문이다. 오히려 부정확한 정보와 소문이 소셜미디어를 타고 확산하는 가운데 일반인의 불안을 가중시킬 우려가 있다.

마지막으로 트라우마 등 정신건강에 대해서도 많은 중요한 논의가 있었다. 특히 위험 위기 상황에서 사회적 지지는 이해관계자 사이에서도 중요한 이슈이며 이에 대한 중재(intervention)와 평가(evaluation)는 중요한 연구과제라고 생각한다. 다만 사회적인 지지가 있었는지의 여부 정도가 아니라 사회적 지지의 대상이 어떤 내용을 얼마나 지지받았는지, 그 내용이 정보적(informational)인지 감성적(emotional)인지, 그 내용이나 형식에서 어떤 것이 만족스러웠는지 등 더 세부적인 내용에 대한 연구가 절실하다.

다양한 공중보건 위기 상황은 앞으로도 계속 닥칠 것이다. 이에 대한 대비는 단순히 공중보건과 의료 전문가만의 몫이 아니라 위험 소통 전문가가 함께 협력할 때 실제 위험과 지각된 위험에 적절히 대응할 수 있을 것이며, 위험평가, 위험관리, 위험 소통의 세 박자가 잘 맞아떨어지는 위험 통제(risk governance) 시스템을 갖출 수 있을 것이다.
